# A Comparison of Phenylephrine and Norepinephrine Infusions in Preventing Hypotension After Subarachnoid Block in Caesarean Section: A Randomized, Double-Blind Study

**DOI:** 10.7759/cureus.82176

**Published:** 2025-04-13

**Authors:** Omer M Mujahid, Mamta Sinha, Mayank Kumar, Subrata K Singha, Monica Khetarpal, Rashmi Dubey

**Affiliations:** 1 Cardiac Anesthesia and Critical Care, All India Institute of Medical Sciences, New Delhi, New Delhi , IND; 2 Anaesthesiology, All India Institute of Medical Sciences, Raipur, Raipur, IND

**Keywords:** caesarean section, hypotension, norepinephrine, phenylephrine, subarachnoid block

## Abstract

Background

Prophylactic vasopressor infusion is commonly recommended during cesarean section under spinal anesthesia to prevent hypotension. Norepinephrine is gaining recognition as a viable alternative to phenylephrine in obstetric anesthesia, primarily due to its lower propensity to cause reflex bradycardia. This study compares the efficacy and safety of prophylactic intravenous infusions of norepinephrine and phenylephrine, administered at varying infusion rates, on maternal hemodynamics and neonatal outcomes during cesarean delivery.

Methods

In this prospective, randomized, double-blinded study, 102 parturients of ASA physical status 2, undergoing cesarean section under Lucas Grade 1 and 2, were randomized into two groups to receive prophylactic manually adjusted intravenous infusions of norepinephrine or phenylephrine. The primary objective was to compare the efficacy of norepinephrine and phenylephrine in preventing post-spinal hypotension. The safety of these two drugs with respect to the neonatal outcome and maternal bradycardia, nausea, and vomiting was the secondary objective.

Results

Ninety-five parturients were analyzed, and the incidence of hypotension was similar between the two groups (27.08% vs. 21.28%, p = 0.509). However, the phenylephrine (PE) group had a significantly higher incidence of bradycardia compared to the norepinephrine (NE) group (19.15% vs. 0%, p = 0.001). The incidence of nausea and vomiting was comparable in groups NE and PE ( 4.17% vs. 10.64%, p = 0.268). The number of manual physician interventions was similar between the groups (27.08% vs 21.28%, p = 0.509). Neonatal cord blood ABG parameters were comparable between the groups.

Conclusion

Prophylactic intravenous infusions of phenylephrine and norepinephrine showed similar efficacy in preventing hypotension. However, the use of norepinephrine did not cause bradycardia as compared to phenylephrine.

## Introduction

Subarachnoid block (SAB) is a standard method of anesthesia for elective cesarean deliveries [1]. SAB up to the level of T4 is necessary for cesarean birth, which inadvertently causes thoracolumbar sympathetic outflow blockade, leading to a decrease in systemic vascular resistance (SVR) and hypotension. This is further compounded by the aortocaval compression in the parturient [2].

SAB-induced hypotension can cause dizziness, nausea, vomiting, and decreased levels of consciousness in the mother, and the decrease in uteroplacental perfusion can lead to fetal hypoxia, fetal acidosis, and poor neonatal outcomes [3]. The duration of hypotension is more important than the degree of hypotension in affecting neonatal outcomes [4]. Counteracting the decrease in systemic vascular resistance with a prophylactic vasopressor infusion is now a fundamental practice in patients receiving SAB for cesarean deliveries [5]. Many vasopressors such as phenylephrine, ephedrine, and, recently, norepinephrine have been used for prophylaxis against post-spinal hypotension (PSH) [5,6].

Phenylephrine, which is a potent alpha-1 agonist with no beta receptor activity in clinical doses, has long been propagated as an ideal drug for prevention and treatment because of its potency, relative safety to the fetus, and rapid onset of action [5]. However, phenylephrine, whether administered as a bolus or through continuous infusion, can cause baroreceptor-mediated reflex bradycardia, limiting its use in parturients with cardiac comorbidities or in situations where fetal compromise is already present [7].

Norepinephrine, recently introduced in obstetric anesthesia, has a predominant alpha receptor agonist activity and weak beta receptor agonist activity. It has been associated with lower risks of bradycardia [8]. However, comparative studies of these two drugs as manually titrated, continuous infusions are limited. The primary objective of this study was to compare the efficacy of phenylephrine and norepinephrine administered as a variable, manually titrated continuous infusion in preventing hypotension following subarachnoid block during elective cesarean deliveries. Our secondary objective was to compare the side effects of both drugs in terms of neonatal outcomes like fetal acid-base balance and maternal outcomes, including bradycardia, nausea, and vomiting.

## Materials and methods

After obtaining clearance from the Institute Ethics Committee, All India Institute of Medical Sciences, Raipur (753/IEC-AIIMSRPR/2019) and registration with the Clinical Trials Registry of India (CTRI/2020/03/023761 dated 04/03/2020), prospectively, parturients undergoing caesarean section were recruited for the study. This was a randomized, double-blinded, parallel group-controlled clinical study conducted between 10/03/2020 and 15/06/2021. The study followed all the principles of the Declaration of Helsinki. 

Pregnant women planned for caesarean section (Lucas grade 3 and 4) were screened in the pre-anesthetic area for systemic medical and surgical illnesses. The study included parturients aged 18 to 35 years, with a singleton pregnancy of at least 37 weeks' gestation, classified as American Society of Anaesthesiologists (ASA) physical status II, without any systemic illnesses, and those with Lucas grade 3 or 4 urgency for a Caesarean section. Written informed consent was obtained from them.

Patients who did not qualify for spinal anesthesia- patients with spinal deformities and parturients in whom there was a diagnosed fetal compromise were excluded from the study. Parturients with pregnancy-induced complications like gestational hypertension, gestational diabetes mellitus, abruption placenta, and parturients of height less than 140 cm and more than 170 cm were also excluded from the study. Parturients with failure of subarachnoid block and those converted to general anesthesia were excluded from data analysis.

Patients were randomly assigned to one of two groups, PE (phenylephrine) or NE (norepinephrine). In the PE group, patients received phenylephrine infusion starting at a rate of 25 mcg/min, with phenylephrine hydrochloride diluted to a final concentration of 50 mcg/ml. In the NE group, Norepinephrine bitartrate was diluted to achieve a final concentration of 4 mcg/ml and patients received norepinephrine infusion starting at a rate of 2 mcg/min. The drug volumes in both infusions and the starting infusion rates were the same in both groups.

Randomization was done using computer-generated random numbers (online from researchrandomiser.com). Allocation concealment was maintained using sequentially numbered, opaque, sealed envelopes. Each envelope contained specific instructions for drug preparation and was opened by a nurse not involved in the management of the patient. The nurse prepared the assigned vasopressor and handed it over to the anesthesiologist responsible for intra-operative care, thereby ensuring that the anesthesiologist remained blinded to group allocation.

In the operating room, after connecting standard ASA monitors (electrocardiography, SpO2, non-invasive blood pressure), under aseptic precautions, in sitting position, SAB was given with 2.2 mL of 0.5% bupivacaine (heavy) at L3-L4/L4-L5 intervertebral space using a 25G Quincke’s needle. In patients with a height of less than 150 cm, 2.0 ml of the drug was used. Vasopressor infusion was started as soon as the SAB was given, based on the group of the patient. Heart rate and non-invasive blood pressure were recorded at 1-minute intervals until the delivery of the baby. Vasopressor infusions were manually titrated along with vasopressor boluses for hemodynamic management. 

If the patient developed hypotension, where hypotension was defined as a fall in systolic blood pressure by more than 20% of baseline, the infusion rate was increased by 20%, and a bolus dose of 25 mcg of phenylephrine was administered in both groups. In the case of reactive hypertension, where there was more than a 20% increase in systolic blood pressure from baseline, the infusion was stopped. Bradycardia was a fall in heart rate to less than 60 beats per minute. Intravenous atropine 0.6 mg was given if the heart rate was less than 50 beats per minute (bpm) or if associated hypotension was present. Vasopressor infusion was stopped as soon as the baby was delivered. A cord blood sample from the umbilical artery of the neonate was taken and sent for umbilical blood gas analysis (pH, pO2, pCO2, HCO3).

Sample size

The sample size was determined based on a prior study by Allen et al., which reported a 30% incidence of hypotension in the phenylephrine group [9]. Assuming an absolute difference of 20% in the incidence of hypotension between the two groups, a power of 80%, and a significance level of 95%, the calculated sample size was 45 participants per group. To account for potential dropouts or loss of participants during the study, the sample size was increased to 51 participants per group, resulting in a total of 102 participants.

Data collection was performed by retrieving hemodynamic parameters recorded and stored in the patient monitor. Statistical analysis was performed using the Statistical Package for the Social Sciences (SPSS) for Windows, version 21 (IBM Corp., Armonk, NY). Data that followed a normal distribution were presented as mean ± standard deviation, and comparisons between the two groups were conducted using an independent t-test. For data that did not follow a normal distribution, medians (with quartiles) were used to represent the values, and the Mann-Whitney U test was employed to compare differences between the two groups. The Chi-square test or Fisher’s exact test was employed to evaluate associations between categorical variables. A p-value of <0.05 was considered statistically significant.

## Results

**Figure 1:**
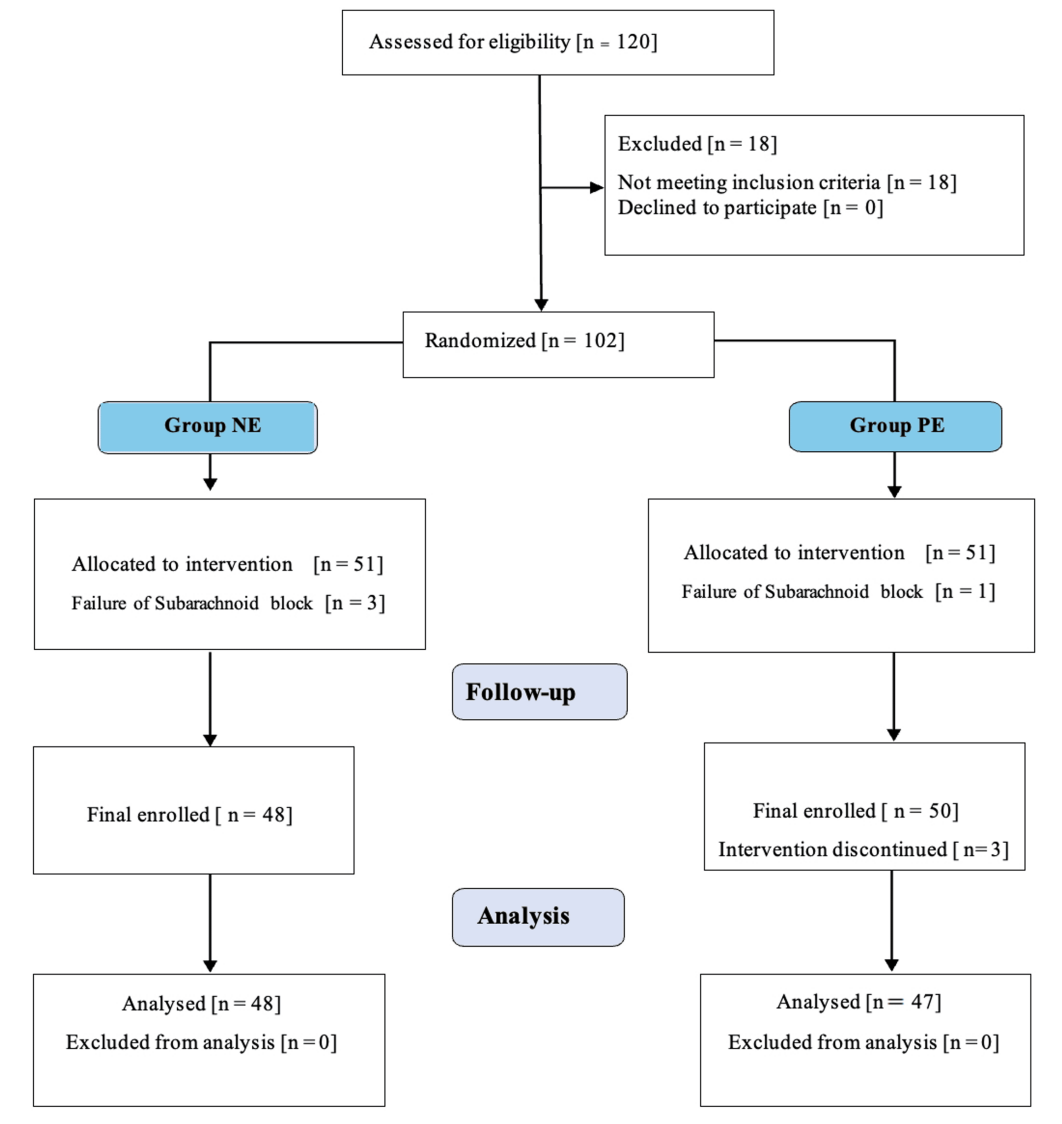
CONSORT diagram CONSORT: Consolidated Standards of Reporting Trials, NE: Norepinephrine, PE: Phenylephrine.

In the present study, a total of 120 parturients were screened for eligibility, and 102 were randomized equally into two groups. Failure of spinal anesthesia was observed in four patients, and so they were excluded from data analysis. In three patients, the intervention was discontinued, and hence, they were excluded, so the final data was analysed for 95 patients (Figure 1).

The two groups were comparable regarding age and anthropometric parameters (weight, height and body mass index) with no significant differences (p >0.05) (Table 1).

**Table 1:** Demographic data, operative data, and baseline characteristics. Data are presented as Mean ± Standard Deviation NE: Norepinephrine, PE: Phenylephrine, BMI: Body Mass Index, SAB: Subarachnoid Block.

	Group NE (n=48)	Group PE (n=47)	p-value
Age (years)	28.21 ± 3.78	27.53 ± 3.88	0.392
Height (m)	1.62 ± 0.05	1.63 ± 0.05	0.863
Weight (kg)	67.6 ± 7.38	65.57 ± 6.23	0.151
BMI (kg/m^2^)	25.59 ± 2.26	24.8 ± 2.41	0.101
SAB to delivery of the foetus time (in minutes)	11.86 ± 3.90	10.76 ± 3.67	0.162
Baseline Heart Rate (beats per minute)	102.54 ± 17.35	102.68 ± 17.3	0.969
Baseline Systolic Blood Pressure (mm of Hg)	121.27 ± 14.7	123.38 ± 9.61	0.409

The incidence of post-subarachnoid block hypotension was comparable in both groups (27.08% vs. 21.28%, respectively) (p =0.509). In the PE group, nine out of the 47 study participants had bradycardia. This was significantly higher than the NE group (19.15% vs 0%, respectively, p value=0.001). The incidence of reactive hypertension between the groups was non-significant: 4.17% in the NE group vs 0% in the PE group (p=0.495). The incidence of nausea and vomiting between the groups was comparable and non-significant (p=0.268); however, it was less in the NE group than in the PE group (4.17% vs 10.64%, respectively). The number of parturients needing manual interventions in both groups was comparable: 27.08% in the NE group versus 21.28% in the PE group (p=0.509) (Table 2).

**Table 2:** Maternal outcomes Data are presented as numbers and frequency (%). *p < 0.05 denotes statistical significance. NE: Norepinephrine, PE: Phenylephrine.

	Group NE (n=48)	Group PE (n=47)	p-value
Post Spinal Hypotension	13 (27.08%)	10 (21.28%)	0.509
Bradycardia	0 (0%)	9 (19.15%)	0.001*
Reactive hypertension	2 (4.17%)	0 (0%)	0.495
Nausea/Vomiting	2 (4.17%)	5 (10.64%)	0.268
Number of patient required manual Intervention	13 (27.08%)	10 (21.28%)	0.509

Umbilical artery blood was analysed for pH status, pO2, pCO2 and HCO3, and values of these parameters were compared. In both groups, the pH values were comparable, and the mean pH ranged between 7.34 ± 0.05. Umbilical artery pO2 values in the NE group were higher than in the PE group, but values were comparable in both groups (p=0.1) (Table 3).

**Table 3:** Neonatal outcomes: cord blood ABG parameters Data are presented as Mean ± Standard Deviation NE: Norepinephrine, PE: Phenylephrine, ABG: Arterial Blood Gas.

	Group NE (n=48)	Group PE (n=47)	p-value
pH	7.34 ± 0.06	7.34 ± 0.04	0.501
pO2 (mmHg)	24.04 ± 5.18	22.8 ± 5.82	0.1
pCO2 (mmHg)	45.02 ± 5.69	43.69 ± 3.03	0.161
HCO3 (mEq/L)	22.3 ± 3.42	21.89 ± 2.22	0.25

## Discussion

The results of our study demonstrate that prophylactic infusions of both norepinephrine and phenylephrine effectively controlled maternal blood pressure during cesarean delivery; however, a lower incidence of bradycardia was observed in the NE group as compared to the PE group. Our results align with previous studies like the study by Kee et al., where the authors used computer-controlled infusions of NE and PE during cesarean delivery [8]. Their results showed norepinephrine to have similar efficacy as phenylephrine in maintaining maternal blood pressure with a higher heart rate. A similar study conducted by Hasanin et al. utilized manually adjusted variable infusions of norepinephrine and phenylephrine to prevent post-spinal anesthesia hypotension [10]. Their findings demonstrated that both vasopressors were effective in maintaining maternal blood pressure during cesarean deliveries.

Phenylephrine, a selective alpha-1 adrenergic agonist, has long been considered the vasopressor of choice in obstetric anesthesia [11], replacing ephedrine due to its potential effects on fetal acid-base balance [12]. However, the absence of any beta-mimetic effect and baroreceptor activation can lead to bradycardia, which can lead to a fall in the maternal cardiac output and adversely impact the uteroplacental circulation [6,13].

This concern is supported by the significantly higher incidence of bradycardia in the PE group than in the NE group, where bradycardia was defined as a heart rate less than 60 bpm. Norepinephrine, an alpha and beta-agonist with potent alpha-mimetic action and weak beta-agonist effect, has less propensity than phenylephrine to cause bradycardia, probably due to the positive chronotropic activity of its beta-mediated action. Our findings are consistent with the study by Kee et al., who reported a higher heart rate and, subsequently, greater cardiac output in the norepinephrine group compared to the phenylephrine group [8]. However, the study by Feng et al., which employed non-invasive methods to measure cardiac output, reported that cardiac output was maintained in both the vasopressor groups [14].

Similarly, Singh et al. reported a higher incidence of bradycardia in the PE group (43.3%) compared to the NE group (20%), although the difference was statistically nonsignificant [15]. Despite these mixed results, the tendency towards lower bradycardia in the NE group highlights norepinephrine as an attractive choice of vasopressor in obstetric anesthesia. In terms of dosing, Allen et al. conducted a dose-finding study comparing four different doses of phenylephrine infusions during cesarean section and found dose ranges of 25 mcg/min and 50 mcg/min to be most suitable [9]. We similarly used a starting infusion dose of 25 mcg/min in our study. 

Mohta et al. [16], reported a potency ratio of phenylephrine to norepinephrine as 11:1, while Kee et al. [17], in a dose-response study, determined the ratio to be 13:1. In our study, the concentrations of phenylephrine (50 mcg/mL) and norepinephrine (4 mcg/mL) were in a ratio of 12.5:1. This ratio proved equipotent in preventing hypotension, as evidenced by the comparable and statistically non-significant incidence of hypotension between the two groups.

Another important consideration in obstetric anesthesia is the incidence of nausea and vomiting. Sympatholysis-induced hypotension results in reduced cerebral perfusion, leading to transient ischemia of the brainstem, which activates the vomiting centers and triggers nausea and vomiting. Additionally, the reduction in systemic vascular resistance (SVR) also decreases splanchnic perfusion, leading to the release of emetogenic substances. These two factors contribute to nausea and vomiting following spinal anesthesia [18]. One of the secondary objectives of our study was to assess the incidence of nausea and vomiting. The results were comparable and non-significant; however, we observed that the incidence of nausea and vomiting was lower in the NE group compared to the PE group.

In terms of fetal outcomes, analysis of the umbilical artery blood in our study showed no incidence of fetal acidosis (pH < 7.2), with mean pH values ranging from 7.34 ± 0.05. The pH values were comparable between the two groups, with no significant difference. Our findings are consistent with the study by Singh et al. [14], which compared phenylephrine (50 mcg/min) and norepinephrine (2.5 mcg/min) and reported statistically non-significant differences in the mean umbilical arterial blood pH between the two groups. In our study, although the umbilical artery pO2 values in the NE group were higher than those in the PE group, the values were still comparable between both groups.

Vasopressors are conventionally administered through a central venous catheter, and administering them through a peripherally placed venous catheter is said to carry a risk of extravasation and its sequelae. However, recent studies have shown that when administered at low doses through peripheral catheters, the risk of complications is minimal [19,20].

Study limitations

Our study had some limitations. It is a single-center study, and only healthy parturients who underwent elective cesarean section were included in our study. Also, we did not measure cardiac output, and blood pressure recordings were done non-invasively.

## Conclusions

In conclusion, prophylactic infusions of both phenylephrine and norepinephrine were found to be effective in preventing hypotension following spinal anesthesia during elective cesarean sections. However, the use of norepinephrine did not cause maternal bradycardia compared to phenylephrine and both the vasopressors demonstrated comparable neonatal safety profiles. These findings suggest that norepinephrine may be considered a potential alternative to phenylephrine in obstetric anesthesia, though further studies are warranted to confirm these observations.
